# Effect of socioeconomic conditions on health care utilization in marital violence: a cross-sectional investigation from the Japanese Study on Stratification, Health, Income, and Neighborhood

**DOI:** 10.1186/s12939-017-0528-8

**Published:** 2017-02-28

**Authors:** Maki Umeda, Norito Kawakami, Elizabeth Miller

**Affiliations:** 1 Graduate School of Nursing, St. Luke’s International University, 10-1 Akashi-cho, Chuo-ku, Tokyo, Japan; 2 Department of Mental Health, Graduate School of Medicine, The University of Tokyo, 7-3-1 Hongo, Bunkyo-ku, Tokyo, Japan; 3 Division of Adolescent and Young Adult Medicine, Pediatrics, University of Pittsburgh School of Medicine, 3420 Fifth Ave, Pittsburgh, PA USA

**Keywords:** Intimate partner violence, Access to health care, Health inequalities, Socioeconomic factors, Employment, Social support, Health literacy, Mastery, Japan

## Abstract

**Background:**

The health-care-seeking process while experiencing marital violence can be significantly influenced by one’s socioeconomic status, which limits the availability of resources and opportunities for accessing those resources. This study exploratorily examined the effects of socioeconomic factors on the association between marital violence and health care utilization in Japan.

**Methods:**

Cross-sectional data on 2,984 male and female community residents aged 25 to 50 years was obtained from the first wave of Japanese Study of Stratification, Health, Income, and Neighborhood (J-SHINE) conducted between 2010 and 2011. Multiple logistic regression analysis was conducted to examine the association between marital violence and health care utilization. Interaction terms were used to examine the moderating effect of educational attainment, household income, and employment status on the association. Mediation analysis was conducted to estimate the magnitude of mediating effects of mastery, social support, and health literacy in relation to the moderating effect of socioeconomic factors.

**Results:**

Health care utilization in Japan was more prevalent among those who experienced marital violence (69.4 vs. 65.1%). The association between marital violence and health care utilization differed by employment status at a 0.10 level, while educational attainment and household income did not have substantial influence on health care utilization in the presence of marital violence. None of the psychosocial resources (mastery, health literacy, instrumental support, and informational support) explained the differential association by employment status.

**Conclusions:**

This study highlights the increased health care needs of those experiencing marital violence in Japan. The health care needs of the unemployed are potentially unmet in the presence of marital violence. Removing barriers to health care experienced by the unemployed may be an effective strategy for connecting survivors to needed supports and care.

## Background

Intimate partner violence in a marital relationship, “marital violence,” is defined as a pattern of behaviors that harms the psychological, physical, sexual and social well-being of the perpetrator’s current or former spouse [1]. A national survey in Japan found that 23.7% of women and 16.6% of men have experienced physical, psychological, and/or sexual violence by their spouses, and that 56.7% of them had never disclosed their experience of marital violence [2]. Unlike casual and less formal intimate relationships, marriage usually involves cohabitation, sharing of financial properties, dependency on spousal income, and having children. In Japan, a strong public sanction against divorce can create further complexity and barriers to seeking care for those experiencing marital violence [3].

Although health care providers are the most frequently contacted professionals due to the poor physical and mental health that typically accompanies marital violence [4–6], health care may be underutilized in the presence of marital violence. Seeing health care providers may result in disclosing the marital violence to others outside of the family, which could cause negative consequences, such as feelings of shame and embarrassment, stigmatization, disruption to family, escalation of marital violence, and being reported to the police and social services [3, 7–11]. One of the factors that can affect care-seeking behaviors in marital violence is one’s socioeconomic condition. For example, those who have more financial resources, working potential, or stable jobs may have greater freedom in conceptualizing certain situations as unacceptable or intolerable and seek external support because of their resources and options for actions [8, 12]. Educational attainment, household income, and employment status are among the socioeconomic conditions that may determine the resources available to facilitate problem-solving in marital violence. However, thus far, few studies have examined the effect of socioeconomic conditions on health care utilization in marital violence [13–15] and none have investigated the effects of employment status.

Understanding how socioeconomic conditions affect care-seeking in marital violence is crucial for extending existing theories and developing effective intervention strategies. Psychosocial resources can be the factors that influence care-seeking behaviors, interacting with marital violence and socioeconomic conditions. A sense of control and self-efficacy (mastery) were often lowered in the experience of marital violence [16, 17], which could be aggravated by low educational attainment, poverty, and unemployment [12]. On the other hand, higher educational attainment, higher household income, and being in employment likely strengthen one’s sense of effectiveness through enhanced abilities, extended social contacts, and participation and success in the labor market [18–20]. The enhanced mastery may promote care-seeking in marital violence, owing to the positive self-appraisal of one’s ability and easier access to relevant information (health literacy) using this ability [12].

Another possible psychosocial factor is social support. Support from family, friends, and colleagues for dealing with marital violence includes financial assistance, the offering of shelters, giving information and advice, and accompanying them to professional services [12, 21, 22]. The availability of these support systems is affected by socioeconomic conditions. Those having a better socioeconomic condition may have better access to a network of people with material resources to offer and with information, skills and social contacts that can be used for dealing with marital violence effectively [12]. However, the contribution of these psychological resources on health care utilization in marital violence has not been empirically examined.

This study aimed at exploratorily examining the effects of socioeconomic factors on the association between marital violence and health care utilization patterns among Japanese community residents. Our hypothesis was that those experiencing marital violence were more likely to use health care compared to those who were not experiencing marital violence, independent of their socioeconomic conditions. The second hypothesis was that those with lower educational attainment, lower household income, and those without employment were less likely to use health care in the presence of marital violence. We also examined whether the moderating effects of socioeconomic conditions could be explained by difference in levels of psychosocial resources, i.e. mastery, health literacy, and social support. In Japan, universal health coverage lowers barriers in accessing health care among general population. This condition would create an ideal setting to examine the other mechanisms by which marital violence interacts with socioeconomic factors to impact care seeking patterns of those experiencing such violence.

## Methods

### Study design and sample

Our research hypotheses were tested using data from the Japanese Study of Stratification, Health, Income, and Neighborhood (J-SHINE) [23]. We used the first wave of the J-SHINE data collected between October 2010 and February 2011, the most recent data available at the time of this study (August, 2012). The selection of survey sites was based on the cooperation of local governments, and the data were collected in four municipalities in and around the Tokyo metropolitan area. Survey participants were randomly selected from voter registration lists. The age range of participants was from 25 to 50 years old at the time of recruitment, which spanned young adulthood to middle-age, which allowed us to investigate the association of one’s socioeconomic conditions with health in the working age population. The questionnaire was self-administered with a computer-assisted personal interview program. The total number of participants was 4,381 with a response rate of 31.5%.

A total sample of 2,984 participants was used for these analyses, after excluding the data of 1,341 respondents who did not have spouses or common-law partners at the time of data collection, or failed to report their partner status. Fifty-six respondents who did not respond to a set of questions on marital violence, or had missing data on health care utilization were further excluded. Those with missing values were more likely to be unemployed, excluding housewives and househusbands (i.e., individuals who are choosing to work in the home without income, *p* = 0.002, two-sided chi-squired test), and among those with lower educational attainment (*p* = 0.059, two-sided chi-squired test).

## Measures

### Marital violence

Three subscales of the Japanese version of revised Conflict Tactics Scales Short Form (CTS2SF) that correspond to violence were used to measure psychological and physical marital violence that occurred in the past twelve months [24]. We created a dummy variable of marital violence that takes value 1 if a person experienced any marital violence (either victimization or perpetration) in the past twelve months, and zero if a person experienced no marital violence in the same period of time. The use of dummy variable was due to the low prevalence of those who experienced marital violence twice or more in the present study (2 to 3% in physical violence, and less than 1% in injury). It was also because most of those who experienced one type of violence experienced other types in previous studies, and thus we were not able to combine frequency information across different types of violence as suggested by the original developer of this scale [25]. The types of marital violence included were psychological violence (insulting, swearing, shouting, and threatening), physical violence (pushing, shoving, slapping, punching, kicking, and beating), and injury (sprains, bruises, small cuts, pain, and injuries that required medical treatment). Two types of Cronbach alpha were calculated to examine internal consistency of CTS2SF in the present study: raw alpha based on covariance of items and standardized alpha based on correlation of items. When the variances of the items vary widely, a coefficient of raw alpha will be low. (raw Cronbach alpha = 0.77; standardized Cronbach alpha = 0.80). The item related to psychological violence that we used in the present study was slightly different in Japanese expression from the authorized version, because it was revised according to the suggestion of the original author of CST2SF for the equivalence with the original English scale after the administration of J-SHINE [24]. The agreement of the dichotomized composite variables for the pre-revised items and the revised items was high (0 = *no severe psychological aggression either by respondent or partner* and 1 = *any severe psychological aggression by respondent and/or partner*): Kappa coefficient = 0.71 (SE = 0.07), Yule Q = 0.99 (SE = 0.01).

### Health care utilization

One item asked about the experience of receiving outpatient care in the past twelve months. We excluded visits to a health care facility for general health check-ups aiming at screening for socially prevalent diseases (such as colonoscopy and lipid screening), which were often provided by employers or government on a routine basis, health consultations that offered health information but neither diagnosis nor clinical treatment, immunizations, and dental treatments. We also did not include any admissions to hospital as these would reflect a serious health problem including potentially a severe injury that would fall under universal insurance coverage, rather than care-seeking behaviors that may be influenced by socioeconomic factors.

### Socioeconomic factors

Educational attainment was defined by the final educational institutions in which the person was enrolled: junior high school and high school, two-year college and vocational college, and university or higher. Household income was measured by fifteen income bands. A median for each band was divided by the root of the number of household members, and categorized into “low”, “average” and “high.” Employment was measured by one item that asked about current employment status; a dichotomous variable was created by coding “being employed” and “taking leave” as “employed”, and the remainder as “not employed.” Those working in the home without an income by choice (e.g., housewife) were defined as “not employed.”

### Psychosocial resources

#### Mastery

Four items on mastery were derived from a personal mastery scale used in a large-scale community survey in Japan and the United States [26, 27]. The scales asked how strongly the respondents agreed or disagreed with the following statements about themselves: “*I can do just about anything I really set my mind to*”; “*When I really want to do something, I usually find a way to succeed at it”*; “*Whether or not I am able to get what I want is in my own hands*”; and “*What happens to me in the future mostly depends on me.*” All items were based on a seven-point Likert scale (from 1 = *totally applicable* to 7 = *never applicable*). For the current analysis, scores were reversed and summed up for a total score (raw Cronbach alpha = 0.77; standardized Cronbach alpha = 0.77), so that higher scores reflected greater mastery.

#### Health literacy

Respondents indicated their level of agreement to statements about their ability to gather and utilize information on health [28]: “I can seek information from various sources, such as newspapers, books, TV, and the internet”; “I can extract relevant information from various sources”; “I can understand the information and communicate it to others”; “I can consider the credibility of the information”; and “I can make decisions for my improving health based on the information that I’ve got.” All items were measured by a five-point Likert scale (from 1 = *totally disagree* to 5 = *strongly agree*), and the scores from each item were summed to obtain a total score (raw Cronbach alpha = 0.84; standardized Cronbach alpha = 0.84).

#### Social support

Two types of social support were included in the current analysis: instrumental and informational supports. In J-SHINE, each type of support was measured by one item: “How much practical support do the following persons give you when you need some help in your daily life?” (instrumental); and “How much do the following people give you helpful guidance when you have a problem or are in a trouble?” (informational). Respondents were asked to choose one response option from a five-point Likert scale (1 = *a lot*, 2 = *some*, 3 = *little*, 4 = *never*, and 5 = *not applicable*). Each item asked about one of the five sources of support: spouse/partner, other co-residing family members, non-co-residing family members or relatives, neighbors, and friends. For the current analysis, support from spouse/partner was excluded given the strong negative correlation between marital violence and support from spouse/partner. Instead we were interested in support from sources other than their partners. The scores were reversed, and the reversed scores of each source of support were totaled for each type of support, with higher scores indicating greater perceived support.

Mastery, health literacy, instrumental support and informational support were re-coded into three categories with approximately equal frequency distribution so they could be used in logistic regression analysis and OLS liner regression analysis. The total scores of mastery were centered to the mean by rescoring them into the difference from the mean, because it had a U-shape association with marital violence.

### Covariates

All statistical models included the following covariates: gender, age, number of children, and access to health care. Difficulty in accessing health care was measured in terms of physical inaccessibility due to either (1) the absence of health care facilities near one’s house, and/or (2) the lack of transportation to health care facilities. The survey area had four categories, each of which corresponded to a municipality of residency.

### Statistical analysis

For the descriptive analysis, we compared the prevalence of health care utilization and marital violence in the past 12 months by socioeconomic factors and gender. The independent association of marital violence with health care utilization was further examined using multiple logistic regression analysis while adjusting for socioeconomic factors and control variables (model 1). We investigated the muti-collinearity in this logistic model by variance inflation factor (VIF) using regression analysis. The VIF of each coefficient was between 1.0 and 1.32, and the mean VIF was 1.17. Thus, we presumed that the effect of correlation among the independent variables was not substantial enough to distort the estimation.

The moderating effects of socioeconomic factors were examined by adding the interaction terms of marital violence with educational attainment, household income and employment status simultaneously into the multiple logistic regression model (model 2). We further added an interaction term between marital violence and gender to this model to see if the association between marital violence and health care utilization would differ by gender. The overall association between health care utilization and each variable was assessed using the likelihood ratio test for a Type III analysis of effect, which examined a null hypothesis that all individual coefficients of the variable set were equal to zero. Considering the risk of detecting spurious interactions by raising the Type I error rate [29], the level of significance for the interaction terms was set at 10% in this study.

**Fig. 1:**
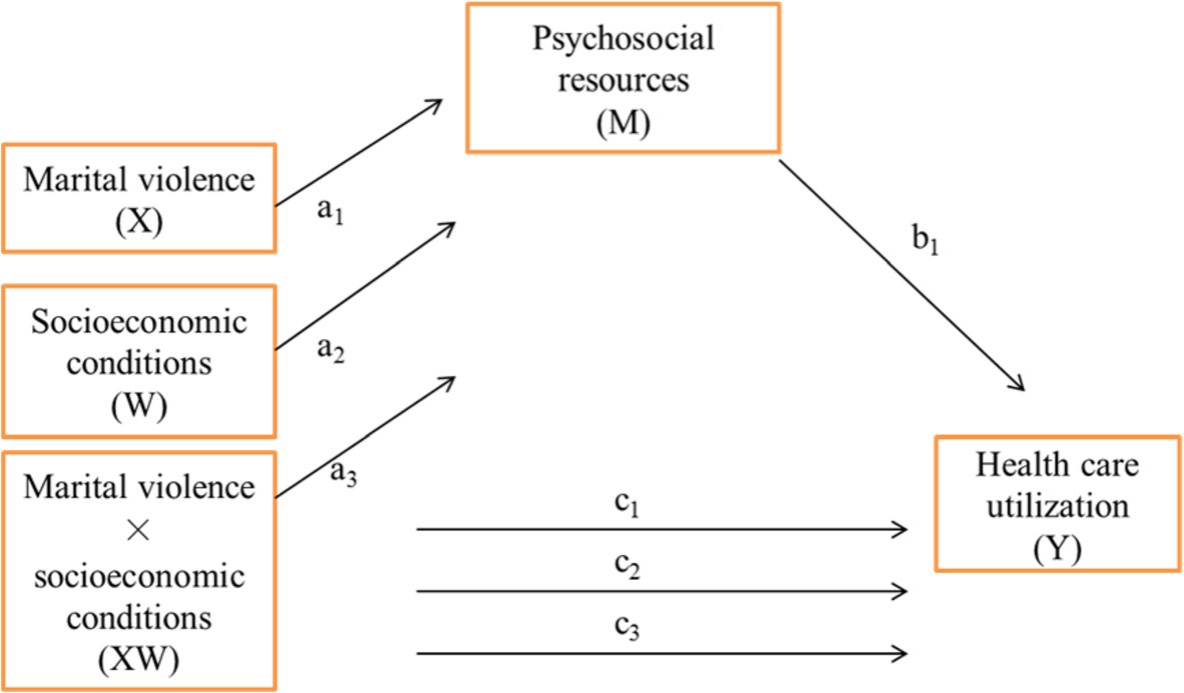
Indirect effect of psychosocial resources (M) between the moderated association between marital violence and health care utilization by socioeconomic conditions (XW)

Mediation analysis was conducted to calculate how much of the moderating effect of socioeconomic factors could be explained by the extent of psychosocial resources respondents had access to. The analysis was conducted using a SAS macro developed by Hayes [30], which adopts a path analysis framework for moderation and mediation analysis. The mediating effect of psychosocial resources was estimated by two regression models; a mediating variable, psychosocial resources, as a dependent variable (ordinary least squares linear regression), and an outcome variable, health care utilization, as a dependent variable (logistic regression). The mediating effect was quantified as the coefficient of the interaction term for marital violence and socioeconomic factors (*a*_3_ in Fig. 1) and the coefficient of psychosocial resources on healthcare utilization (*b*_1_ in Fig. 1), calculated by *a*_3_ × *b*_1_. Bias-corrected 95% confidence intervals and standard error (SE) for the indirect effects were computed based on bootstrap estimation with 10,000 replications. Statistical significance was evaluated using 0.05 level two-sided tests, except for that of the interaction terms. All analyses were conducted using the SAS® statistical package (version 9.3, SAS Institute Inc, Cary, NC, USA), and STATA (version 14, Stat Corp, College Station, TX, USA) for the calculation of VIF and post-hot likelihood ratio test.

## Results

The reported twelve-month prevalence of health care utilization and marital violence was 66.3 and 28.0%, respectively (Table 1). Health care utilization was more prevalent among those who experienced marital violence (69.4 vs. 65.1%). Those who had a higher educational attainment and a higher household income also demonstrated a higher prevalence of health care utilization. None of these profiles differed statistically in prevalence of marital violence.

**Table 1:** Characteristics of respondents in marital relationships based on their health care utilization and experience of marital violence in the past 12 months

		Total (N = 2,984)	Healthcare care utilization in the past 12 months (*n* = 1,978)			Any marital violence in the past 12 months (*n* = 834)		
		%^a^	Prevalence %^b^	Χ^2^ (DF)	*p* value	Prevalence %^b^	Χ^2^ (DF)	*p* value
Health care	Utilized	66.3				29.3	5.10 (1)	0.024^*^
	Not utilized	33.7				25.4		
Marital violence	Any	28.0	69.4	5.10 (1)	0.024^*^			
	None	72.1	65.1					
Education	Jr. high/high	27.0	62.4	13.56 (2)	0.001^*^	29.7	2.34 (2)	0.310
	College	31.3	64.7			28.6		
	University	41.7	69.9			26.7		
Household income	Low	26.5	60.7	23.73 (2)	<0.001^*^	30.8	5.36 (2)	0.068
	Average	36.9	66.5			29.5		
	High	36.6	72.4			25.8		
Employment	Employed	77.9	66.8	1.38 (1)	0.239	27.1	3.18 (1)	0.075
	Not-employed	22.1	64.3			30.7		
Gender	Men	44.0	64.3	4.54 (1)	0.033^*^	28.3	0.05 (1)	0.818
	Women	56.0	68.0			27.9		

^a^Column % (percentage of each of the correlates and demographic categories to the total sample)

^b^Row % (percentage of respondents with health care utilization/marital violence in the past 12 months to the total sample in each correlate and demographic category)

*p < 0.05

The likelihood of health care utilization among those experiencing marital violence was 1.36 times higher than that of those not experiencing marital violence (95% CI = 1.12–1.66), independent of socioeconomic conditions (Table 2). The interaction terms between marital violence and gender in this multiple regression model showed that the association between marital violence and health care utilization did not statistically differ by gender (OR = 0.73, 95% CI = 0.49–1.09, *p* = 0.125; reference = men).

**Table 2:** Moderating effect of socioeconomic conditions on the association between marital violence and health care utilization in the past 12 months^a^ (*N* = 2,357)

		Model 1^a^					Model 2^a^				
		OR	95% CI			*p* value^b^	OR	95% CI			*p* value^b^
Marital violence	Any	1.36	1.12	–	1.66	0.002^*^	1.34	0.9	–	2.01	0.146
(MV)	None	1					1				
Education	Jr. high/high	0.82	0.65	–	1.03	0.041^*^	0.89	0.68	–	1.16	0.108
	College	0.76	0.61	-	0.95		0.76	0.59	–	0.98	
	University	1					1				
Household income	Low	0.68	0.53	–	0.88	0.012^*^	0.63	0.47	–	0.84	0.007^*^
	Average	0.8	0.64	–	1.01		0.76	0.58	–	0.98	
	High	1					1				
Employment	Not employed	0.82	0.64	–	1.04	0.107	0.93	0.7	–	1.23	0.607
	Employed	1					1				
Interaction											
Education	MV x Jr. high/high						0.74	0.45	–	1.24	0.456
	MV x some college						0.99	0.61	–	1.59	
Household income	MV x low						1.4	0.83	–	2.37	0.415
	MV x average						1.29	0.8	–	2.09	
Employment	MV x not employed						0.66	0.41	–	1.06	0.086^†^

^a^Adjusted for age, gender, number of children, access difficulty, and survey area. All variables were entered simultaneously

^b^Likelihood ratio test for type-three analysis. DF = 1 for marital violence and interaction term of employment, and DF = 2 for education, household income, the interaction term of education, and the interaction term of household income

^*^ *p* < 0.05, ^†^ *p* < 0.10 for interaction terms

The moderating effects of socioeconomic factors were presented as the interaction terms between marital violence and socioeconomic factors in the multiple logistic regression model (Table 2). The odds ratio of health care use in the presence of marital violence relative to the absence of that violence were significantly lower among respondents who were not employed compared to those in employment at the 0.1 level (OR = 0.66, 95% CI = 0.41–1.06). In this model, the main effect of marital violence did not remain significant (OR = 1.34, 95% CI = 0.90–2.01, *p* = 0.146), which meant that marital violence was not significantly associated with health care utilization when the respondents were out of employment. The results of the type III analysis suggested that and the interaction terms of education (p = 0.456) and household income (*p* = 0.415) were not significant. Three-way interaction among marital violence, socioeconomic conditions, and gender showed that none of the moderation by education, household income, or employment differed between men and women (*p* = 0.697, *p* = 0.724, and *p* = 0.168, respectively).

We conducted mediation analyses to determine how much of the moderating effect of employment could be explained by the presence of psychosocial resources. None of those resources (mastery, health literacy, instrumental support, or informational support) significantly mediated the moderating effect of employment at a 0.05 level, and all the coefficients were close to 0 (data is available upon request).

## Discussion

Among Japanese community residents, health care utilization was more prevalent among those who were experiencing marital violence in the past year than those without such an experience. Although higher educational attainment and higher household income were significant predictors of greater health care use, adjustment for these socioeconomic factors did not alter the strength of the association to any significant degree. These findings highlight the increased health care needs associated with marital violence, and the potential roles health care providers can play in identifying and intervening in marital violence.

We found that the association between marital violence and health care utilization differed significantly by employment status. The odds ratio of health care utilization in the presence of marital violence relative to the absence of that violence were 1.5 times higher among those who were employed compared to those who were not employed. None of the psychosocial resources (mastery, health literacy, instrumental support, and informational support) explained this differential association by employment status.

Marital violence is often accompanied by the control and financial abuse of the partners, where the partners’ permission is often required to go out of the house even for accessing health care [31, 32]. Coupled with the financial dependency on their partners, violence survivors may have been discouraged from contacting health care providers for fear of disclosing their care-seeking to their partners [11]. Increased opportunities for health check-ups at the work place and an increased motivation to maintain health and occupational functioning may also have contributed to the effect of employment. Another possibility is that the employed experienced more severe marital violence than the unemployed [33], and, because of their employment status, had a greater chance to seek health care. However, our post-hoc analysis showed that the unemployed were slightly more likely to experience physical violence and/or injury than psychological violence (OR = 1.05, 95% CI = 1.01–1.05). Thus, the difference in severity of physical violence does not appear to explain the less frequent use of health care among the unemployed. In order to explain how employment status affects the association between health care utilization and marital violence, the context, impact, and health-related correlates of marital violence should be considered in future studies.

Our findings suggest that education and household income did not have substantial influence on health care utilization in the presence of marital violence in Japan. Little influence of education on healthcare use in the context of marital violence is supported by a study in the US [15], while the lack of a moderating effect of household income contradicts previous findings from the US [13] and Canada [14]. In Japan, lower household income may not create additional barriers for health care utilization even in the presence of marital violence, where consumption of health care requires less expense by individual users given universal insurance coverage [34]. It is also possible that the presence of marital violence does not necessarily drive a further increase in health care seeking as living in poverty likely increases health care needs overall [12].

These findings require cautious interpretation given the limitations of this study. First, the temporal order of the study variables cannot be ensured in this explorative study. Health care utilization in the past 12 months was not necessarily triggered by marital violence during the same period due to the lack of information regarding the reasons for seeking health care in J-SHINE. There might be a case in which the employment status changed before and after the marital violence. Considering the fact that we found employment to have only a marginally significant moderating effect, a more rigorous measurement of the variables and a longitudinal or experimental study design are needed in future studies.

Second, we cannot deny the possibility that the moderating effect of socioeconomic conditions that we found were by chance. Our post-hoc likelihood ration test did not find a significant difference in the fit of the models between those models that were unrestricted (model 1) and those restricted by the interaction terms of marital violence and socioeconomic conditions (model 2) (chi-squared = 5.32, df = 5, *p* = 0.379). Although our analytical models were based on theoretical assumptions, future studies may need to specify better models to confirm the moderating effect of the socioeconomic conditions.

Third, the lack of significant moderating effects of educational attainment and household income may be due to the limited response rate of J-SHINE, whose participants had a higher level of educational attainment and a higher household income than the general population in Japan. Among those with relatively high level of educational attainment and high household income, the effect of these socioeconomic factors may have been smaller than that in a more representative sample. Third, we may have failed to capture some potentially distinct differences in health care needs between victims and perpetrators in combining any experiences of marital violence [35]. In the current sample, about 80% of respondents reported both victimization and perpetration. We did not distinguish victimization from perpetration so as to avoid misclassification resulting from our lack of contextual information on the violence reported in this survey [25, 36]. A more detailed assessment of violence is needed in future studies to investigate the specific perpetration/victimization patterns and associations with health care utilization. Forth, sexual violence was not measured in J-SHINE because of the stigma and embarrassment attached to sexual violence in Japanese society which could have evoked negative reactions toward participation in this multidisciplinary study [37]. The exclusion of sexual violence as a factor in this study may have resulted in weakening the association between marital violence and health care utilization.

## Conclusion

These limitations notwithstanding, this study underscores the increased health needs of those experiencing marital violence among Japanese. Our results suggest the potential contributions of heath care providers to the secondary and tertiary prevention of marital violence – opportunities to provide universal education about the impact of marital violence on health, brief harm reduction counseling, and the availability of support services such as advocacy and counseling [38, 39]. It should be noted, however, that only a small proportion of patients disclose the experience of violence to health care providers [2, 40], being afraid of negative consequences, such as feelings of shame and embarrassment, stigmatization, disruption to the family, escalation of the violence, and being reported to the police or social services [7, 8, 10, 11]. Health professionals’ lack of knowledge and confidence in dealing with marital violence and the shortage of time and space for discussing this issue in privacy [7, 10, 11, 41] need to be overcome so that the use of health care becomes a real opportunity for discussing marital violence with all patients and providing meaningful interventions [42]. We also found that those who were not employed were less likely to use health care when they experience marital violence. Health and social professionals need to be aware that the health needs of the unemployed are potentially unmet in the presence of marital violence, and that removing barriers to accessing this health care may assist those experiencing this kind of violence. Public health centers are in a position to play a central role in addressing marital violence among those out of employment in Japan, because they often reach out to the unemployed population through the provision of free health programs and health check-up. How employment status affects the health care utilization of those experiencing marital violence needs further exploration. One approach that could prove promising in future studies would be to employ mixed methodologies that incorporate a qualitative investigation into the way employment status formulates one’s care-seeking patterns in the course of marital violence.

## Abbreviations

CTS2SF: The revised conflict tactics scales short form
J-SHINE: Japanese study of stratification, health, income and neighborhood
