# Erratum to: Effect of socioeconomic conditions on health care utilization in marital violence: a cross-sectional investigation from the Japanese Study on stratification, health, income, and neighborhood

**DOI:** 10.1186/s12939-017-0606-y

**Published:** 2017-06-26

**Authors:** Maki Umeda, Norito Kawakami, Elizabeth Miller

**Affiliations:** 1 Graduate School of Nursing, St. Luke’s International University, 10-1 Akashi-cho, Chuo-ku, Tokyo, Japan; 2 Department of Mental Health, Graduate School of Medicine, The University of Tokyo, 7-3-1 Hongo, Bunkyo-ku, Tokyo, Japan; 3 Division of Adolescent and Young Adult Medicine, Pediatrics, University of Pittsburgh School of Medicine, 3420 Fifth Ave, Pittsburgh, PA USA

## Erratum

After publication of this article [1], it was noticed that the “Availability of data and materials” section is incorrect. The corrected text can be seen below.
